# “The supreme discipline of Nursing”–A qualitative content analysis of nurses' opinions on caring for people eighty years of age and older

**DOI:** 10.1016/j.heliyon.2024.e26877

**Published:** 2024-02-22

**Authors:** Lena Maria Lampersberger, Gerhilde Schüttengruber, Christa Lohrmann, Franziska Großschädl

**Affiliations:** Institute of Nursing Science, Medical University of Graz, Graz, Austria

**Keywords:** Nursing care, Aged care, Geriatric, 80+, Qualitative content analysis, Perceptions, Opinions nursing staff, Nurses

## Abstract

**Introduction:**

Nurses often experience stress and feel under time pressure when working with older people, increasing their job dissatisfaction. Especially people 80 years of age and older often require more complex and a greater measure of care, as the risk of care dependency is higher in this age group. This study was conducted to collect nurses’ experiences and opinions regarding the care of people 80 years of age and older, as well as to learn more about how nurses perceive this care.

**Method:**

We analysed narratives collected in an Austrian nationwide, cross-sectional online study to investigate nurses’ attitudes towards people 80 years of age and older and their perceptions regarding their care (N = 1197). Data were collected from May–October 2021 by using a convenience sampling method. In total, 149 participants filled in the free text field; these texts were analysed using a qualitative content analysis method.

**Results:**

Three main themes emerged from the analysis of the nurses' narratives: (1) ‘opinions on people in need of care’, (2) ‘reputation of nursing profession’, and (3) ‘criticism of current nursing practice’. Most narratives were assigned to the subthemes ‘positive opinions on nursing’, ‘ideal image of nursing’, and ‘shortage of staff’.

**Conclusion:**

Nurses considered their work with people aged 80 years and older to be meaningful and important, but they criticised working conditions which need to be improved. This could be achieved by offering further education and increasing nursing staff. Further research is needed to investigate nurses’ needs and wishes regarding the care of people aged 80 years and older.

## Highlights

• We highlight possible barriers that prevent nurses from working with older people.
• Nurses expressed a wish for more geriatric training.
• The nurses' opinions regarding people in need of care are positive.
• Working conditions need improvement to make geriatric nursing more attractive.

## 1 Background

Sociodemographic changes are expected to result in an overall older world population [1], thus the need for specialised geriatric nurses who deliver high-quality care is also expected to increase due to the growing population of older people [2]. By the age of 80, the risk of care dependency increases two-to threefold [3,4]. Care dependent people are more at risk of experiencing falls and pressure ulcers; have more mobility, vision, and hearing difficulties; are less able to care for themselves, i.e. to perform basic and instrumental activities of daily living; have an impaired cognitive status; and have a poorer emotional health status [3]. The same can be seen in Austria, where the need for care and support increases with age [5] and acute care patients have the highest rate of care dependency [6]. Therefore, the proportion of older people in need of care is the highest in all settings in Austria [[6], [7], [8]]: In acute care, 44.9 % of patients were 60–79 years old, and 24.6 % were 80 years and older [6]. In home care, 74 % of clients were 75 years and older, while 38 % were older than 85 years. 82 % of nursing home residents were 75 years or older, and 50 % were 85 years and older [7,8].

As shown above, older adults age individually and may become care dependent sooner or later. When care dependency, frailty, and multimorbidity occur in a person, it is also called the fourth age. The fourth age is not defined by chronological age but is presumed to occur around the age of 80 [9,10]. Due to the increased risk of care dependency with the age of 80 [3,4], older people are defined as people 80 years of age and older in this study. People in this age group may find themselves confronted with stereotypes from society, like being frail, dependent and constituting a burden on society [11], which are mostly persistent over time [12]. People in this age group may also experience ageism and stigmatisation [13]. Especially people with dementia are stigmatised due to both their diagnosis and their age [14]. Nurses may be desensitised in their view of older people by their frequent contact with multimorbid and care-dependent older adults [15], which can affect nurses’ desire to care for older adults [16].

Providing care for older people is not always a preferred career choice for nursing students and nurses [[17], [18], [19]], and this is especially true for caring for older people in nursing homes [20]. One reason for this is that working with older people is perceived as more difficult due to the higher measure of care and more complex care needed [21]. Previous experiences from clinical practice also influence nurses’ willingness to work with older people, depending on whether their experiences were negative [18,21] or positive [17], and studies have shown that nurses can be more or less willing to work in this field [17,18,21]. On the other hand, nursing students may consider working with older people because they hold a generally positive attitude towards older people [18,19] or towards their care [22]. They may also feel that these individuals need more care and want to take the opportunity to increase their quality of life [21].

Nurses' attitudes towards older people generally influence their willingness to work with this age group [16,17]. Attitudes are defined by Eagly and Chaiken (1993) as a ‘psychological tendency that is expressed by evaluating a particular entity with some degree of favor or disfavor’ [23]. Attitudes consist of different components related to someone's feelings, beliefs, and behaviour towards someone or something. Feelings, beliefs and behaviour, i.e. a person's attitude, can be either positive, neutral or negative [24,25]. These attitudes towards older people can affect the quality of care provision, for example, if the amount of care provided is reduced [26,27]. On the other hand, nurses' positive attitudes towards older adults may increase the quality of care and may have a positive effect on caring behaviours like respect [28]. The international literature reports mixed results regarding nurses' attitudes about caring for older people in need of care, but these can be found in all health care settings and health care providers [29]. One systematic review by Rush et al., 2017 [26] reports that nurses hold both positive and negative attitudes. Examples of positive attitudes included having respect for and patience with older people in need of care and giving them the time and attention they need. Examples of negative attitudes included viewing the older people in need of care as weak, disabled, inflexible, and mentally and cognitively impaired, perceiving their care as a burden, and speaking negatively to or about people in need of care. In Austria, Lampersberger et al., 2023 [30] reported that nurses generally perceive the care of people 80 years of age and older as positive. Nurses regarded this care as a challenging but rewarding task, noting that such care was labour-intensive but that it was also worth the investment in terms of their time and energy. The study findings did not indicate that Austrian nurses consider caring for older people as frustrating or this field of nursing to be an undesirable career choice.

Nurses' attitudes about caring for older people can also influence their relationships with the people in need of care. A systematic review by Riviere et al., 2019 [31] cited two core elements of the nurse-patient relationship which are centrally important for older people: respect and dignity. These elements are characterised by nurses’ caring behaviour and their attitudes. This review indicates that people in need of care may experience nurses as being e.g. aware, kind, and interested in them; in this case, they describe the nurses as attentive, helpful, and empathetic. But people in need of care may also experience nurses (and subsequently describe them) as inattentive, ineffective (e.g. offering only fragmented care), or inhumane (e.g. depriving them of care) [31]. Older people in need of care who feel respected by nurses may also show a higher level of satisfaction with their care [32]. A study by Hall and Høy 2012 [33] shows that nurses perceive caring for older people in need of care as a way of helping them regain dignity; the surveyed nurses indicated that they regarded this as their responsibility. These nurses, however, also described barriers that prevented them from achieving this goal: lack of time and the need to focus exclusively on diagnosis and physical treatment.

When caring for older people, nurses often experience feelings of stress and time pressure, which makes it difficult for them to perform their daily work. Dierckx de Casterlé et al., 2020 [34] report that nurses may feel a sense of failure when providing care or when they can offer only basic instead of high-quality care due to e.g. a lack of time which prevents them from having conversations with patients or addressing more than merely basic hygienic needs. In this study, nurses indicated that they were only able to focus on the physical and visible aspects of care. This can also lead to job dissatisfaction, as seen in a Jordanian study where 68% of the surveyed nurses were not satisfied with their job, i.e. with caring for older people in need of care, mainly because of the working conditions and the low pay [35]. According to a Turkish study, nurses experienced various difficulties when providing care for older people, such as inadequate technical equipment, care problems due to patients' limited physical mobility, administrative difficulties, and insufficient knowledge to properly care for older people [36]. Nurses' views about working with older people can be influenced by the amount of gerontological nursing education they have received and their experience in gerontological nursing [26]. Lampersberger et al., 2023 [30] found that nurses who had worked with older care receivers frequently held more negative attitudes towards people 80 years of age and older. In contrast, Rush et al., 2017 [26] observed that nurses who had more work experience also had more positive attitudes but also reported that the nurses' attitudes were influenced by their work environment (i.e. a lack of resources) and stress. Holmberg et al., 2022 [37] also cited conflicting results: Having frequent interactions with people 80 years of age or older in need of care seemed to negatively influence nurses’ general attitudes, but these interactions were also associated with the development of more positive attitudes about caring for people in this age group.

To ensure high-quality care for older people in need of care, nurses with a positive attitude who respect the care recipient are needed [26,31]. However, people 80 years of age and older have a higher risk of care dependency [3]; thus, they may require more complex care and their care may be perceived more negatively by nurses [38]. Additionally, the nurse-patient relationship [31] and quality of care may be influenced by nurses' negative attitudes [26,27]. International studies that examine nurses' opinions regarding the care of older people (especially those 80 years of age and older) and how they experience this care are rare [26]; and, to our knowledge, there are no data on Austrian nurses. As these older persons are the largest group nurses care for in Austria, as well as in other countries, and throughout various health care settings like inpatient care services or home care [7,8], there is a clear need for well-educated nurses delivering high quality care to older people. However, such data is needed to understand nurses' experiences and opinions on caring for older people and to obtain insights into possible barriers for caring for older adults. The primary aim of the original cross-sectional study, which was published previously [30], was to assess nurses' attitudes towards people 80 years of age and older and how they perceive their care. The nurses surveyed in this study were qualified nurses (QN), specialised nurses (SN) and nursing aides (NA), which is described in more detail in the methods section. These nurses had the opportunity to use a provided commentary field at the end of the questionnaire to report their experiences with and opinions on older adults and how they perceive caring for them. It was not expected to receive many narratives, but as we received a high amount of narratives, we decided to analyse these separately from the quantitative results of the cross-sectional study because the narratives may show results which cannot be analysed and described together with quantitative research data. Therefore, the aim of this study is to analyse nurses’ experiences and opinions regarding the care of people 80 years of age and older and to learn more about how nurses perceive this care.

## 2 Methods

### 2.1 Design

This study had a cross-sectional study design and was conducted as an Austrian nationwide, online survey including structured questions presented in the form of a questionnaire. The survey was designed to assess nurses’ attitudes towards older people aged 80 years and older, to identify influencing factors for these attitudes, and to determine how nurses perceived caring for this age group [30]. In total, 1197 persons completed the questionnaire which also included a comment box at the end which enabled open-ended responses (i.e. narratives) to be collected in order to give participants the opportunity to express their opinion on the topic [39]. Although the option to provide a final comment in such boxes is not usually used (3.6–5.7% [40]), 12.64% of the participants in our sample submitted responses in this box. Due to the high numbers of received answers, we analysed these separately by using a qualitative content analysis method [41]. The Standards for Reporting Qualitative Research (SRQR) guideline was applied within this study [42].

### 2.2 Participants and setting

Nurses working in any setting in Austria were included in this study. These nurses worked as qualified nurses (QN), specialised nurses (SN), or nursing aides (NA). Qualified nurses had completed either a bachelor's degree programme or professional vocational training. Specialised nurses had additionally attended a university course in a clinical speciality like intensive care or palliative care. Nursing aides included assistant nurses who had completed one year of training and nursing assistants who had completed two years of training. In Austria, nursing aides are mainly responsible for delivering basic care and assisting qualified nurses to, for example, deliver therapies, monitor patients' state of health, and with patient education [43]. Therefore, nursing aides spend a significant time with people in need of care and were therefore included in this study. Out of the 1197 participants, 149 submitted written narratives. Nurses in the included sample had a median age of 50 years, with an interquartile range (IQR) of 39–54 years, and had worked 25 (IQR = 12–32) years in health care. Table 1 shows the demographic characteristics of the participants, describes their work environments, and shows how often the participants were in contact with people aged 80 and older.

**Table 1:** Demographic characteristics, work environment, and contact with people ≥80 years of the sample.

(*n*)	Category	Participants who did not provide a narrative		Participants who provided a narrative	
		*n*	%	*n*	*%*
Sex	Female	1037	86.5	149	90.6
Origin	Austrian	1047	92.8	148	90.6
Education	Compulsory education	1037	25.6	145	29
	University entrance qualification	1037	27.2	145	26.9
	Vocational training	1037	17.6	145	21.4
	Bachelor's degree	1037	17.6	145	11
	Master's degree	1037	11.4	145	11.7
	PhD Degree	1037	0.7	145	–
Profession	Qualified nurse	1043	55.3	147	55.1
	Specialised nurse	1043	33.4	147	25.9
	Nursing aids	1043	11.3	147	19
Setting	Acute care	1048	76	149	61.1
	Long-term care	1048	18	149	22.2
	Others	1048	6	149	16.8
Work duty	Clinical	1047	74.9	148	73.6
	Management	1047	18.5	148	18.9
	Others	1047	3.3	148	6.1
	Research	1047	0.9	148	0.7
	Teaching	1047	2.4	148	0.7
Clinical focus	No	1048	22.4	149	19.5
	Yes	1048	77.6	149	80.5
	Others		28.1		30.8
	Surgery		25.3		23.3
	Medical		22.1		17.5
	Neurology		6.4		17.5
	Geriatrics		7.9		9.2
	Psychiatry		7.8		6.7
	Palliative		2.2		6.7
Geriatric/Gerontological education	No	1177	78.5	149	69.1
	Yes	1177	21.5	149	30.9
	Stand-alone course		27,4		47.8
	Part of nursing programme		65		41.3
	Others		1.3		6.5
	Postgraduate training		6.2		4.3
Interaction with older people in need of care ≥80	Daily	1179	63.3	149	69.1
	Weekly	1179	13.7	149	12.1
	Occasionally	1179	12.4	149	11.4
	Never	1179	10.6	149	7.4
Having family/friends ≥80	No	1178	23.2	149	21.5
	Yes	1178	76.8	149	78.5

### 2.3 Data collection

Data were collected in the course of the primary online survey from May to October 2021 during the third COVID-19 wave by using LimeSurvey [44]. A convenience sample of Austrian nurses completed a questionnaire, including the open-ended comment box, in approximately 10 min. Participants were recruited via e-mail and newsletter. Nursing managers and directors in Austrian hospitals, nursing homes, and other health care facilities were sent e-mails with the kind request to forward the invitation to participate in and information about the study to the nursing stuff. The Austrian Nursing Association included the invitation in their newsletter, and the invitation was posted on social media pages (Facebook). Reminders were sent out by the Austrian Nursing Association and via social media to ensure large participation.

### 2.4 Data collection instrument

Primarily quantitative data on the nurses’ attitudes towards older people and on their opinions about their care were collected in this cross-sectional study. Two instruments were used for this purpose: The German version of the Ageing Semantic Differential (ASD) scale [45] and the Perspectives on Caring for Older People (PCOP) scale [46]*.* These quantitative data were published previously [30]. At the end of the questionnaire, participants had the opportunity to provide narrative responses and share their thoughts in a comment box for open-ended comments. This box was introduced with the sentence: “If you have any further remarks/narratives, there is room for them here”. The open text field had no character limit.

### 2.5 Ethical considerations

The Ethics Committee of the Medical University of Graz (EK Number 31–320 ex 18/19) gave their ethical approval for this study. Participants received written information about the study and were asked to provide their consent before completing the questionnaire. Data were collected anonymously, and no conclusions could be drawn regarding the nurses’ identities. Participation in the study was voluntary.

### 2.6 Data analysis

A qualitative content analysis method with an inductive approach was used to analyse data [41]. A qualitative content analysis was chosen instead of a quantitative analysis because it offers a more interpretative approach [47]. We chose Schreier's approach to conduct the qualitative content analysis as this method can be applied when using an inductive approach to analyse data [41]. Before developing the coding frame, relevant passages were selected by one author. To develop the inductive coding frame, the data were structured and categories were generated. To do so, a progressive summarising method described by Mayring 2015 [48] was applied. First, relevant passages were paraphrased. Second, the paraphrases were streamlined and generalised. Third, similar paraphrases were compared, and more general paraphrases were created based on their similarity. Finally, categories and subcategories were defined. These steps were performed for all data by one author. Prior to the pilot phase, the data were segmented into coding units by one author to ensure exclusive coding [41]. In the pilot test of the coding frame, blind coding was carried out by two authors, and 50 (33.6%) documents were subsequently chosen randomly for the pilot test. We pilot-tested 50 documents to ensure that each subcategory was applied at least once. Each coder was handed a codebook with the definition of categories and subcategories, their descriptions, and examples; if needed, decision rules were included [41]. After the selected documents had been coded, the coded segments were discussed, and the coding frame was adapted accordingly. To test the revised version of the coding frame, ten (6.7 %) randomly chosen documents were coded blindly using a revised version of the codebook and discussed afterwards. No further changes to the coding frame were made. In total, 40.3% of the data were blind-coded for which a good interrater agreement was reached; therefore, the remaining data were coded and analysed by one author by using the MXQDA 2020 [49] software. Demographic data were analysed using the software IBM SPSS Statistics Version 28.0.1.0. The quotes reported in this article were translated into English by one author and checked for accuracy using a translator and a bilingual person.

### 2.7 Rigour

The face validity of the data is indicated to be high, as no residual categories were needed. Although the data were not evenly distributed between subcategories, this seems to reflect the corresponding distribution of the themes. The coding frame was pilot-tested, revised, and pilot-tested again to ensure its validity and confirmability, enabling us to reach an 83.65% level of intercoder agreement. The intercoder reliability was calculated using the coefficient Kappa (*κ*_*n*_) as described by Brennan and Prediger 1981 [50]. We were able to reach a substantial to nearly perfect agreement level (*κ*_*n*_ = 0.73–0.87) [51,52] with regard to the main categories of the coding frame. In addition, we sought the expertise of an experienced qualitative researcher throughout the process.

## 3 Results

Three main themes reflected the nurses' experiences with and opinions about caring for people 80 years of age and older: (1) ‘opinions on older people in need of care’, (2) ‘reputation of nursing profession’, and (3) ‘criticism of current nursing practice’.

In addition to the main themes, subthemes were also identified in each main theme. Overall, the narratives could be assigned to three main subthemes: ‘positive opinions of nursing’, ‘ideal image of nursing’, and ‘shortage of staff’ (Table 2). Table 2 also shows the used coding frame including definitions. The subthemes within each main theme are described in more detail below.

**Table 2:** Frequencies of subthemes with definitions.

Main Themes	Subthemes		Definition	Frequency
Opinions about Older People in Need of Care	Neutral Opinions about Older People in Need of Care		Nurses express neutral or neither explicitly negative nor positive opinions about people in need of care over 80 years of age. This category also applies when they report on the opinions of others. These are descriptions of character traits and the behaviour of older persons.	5
	Negative Opinions about Older People in Need of Care		Nurses provide negative narratives about people over 80 years of age. This category also applies when they report on the opinions of others. These are descriptions of character traits and the behaviour of older people.	6
	Positive Opinions about Older People in Need of Care		Nurses make positive narratives about people over 80 years of age. This category also applies when they report on the opinions of others. These are descriptions of character traits and the behaviour of older people.	8
	Nurses' Benefit from Older People in Need of Care		Nurses describe what they can take away and learn from caring for people over 80 years of age or how people in need of care have a positive impact on them.	7
	Resources of Older People in Need of Care	Existing Resources	Nurses talk about the resources available to people over 80 years of age. These resources relate to skills, finances, and family and friends.	3
		Lack of Resources	Nurses talk about the lack of resources of people over 80 years of age. These resources relate to skills, finances, and family and friends.	9
	Dealing with Older People in Need of Care is Difficult or Demanding		Nurses describe that caring for or personally interacting with people in need of care over 80 years of age is difficult or demanding work. This category refers only to factors that affect the people in need of care and not to external factors, such as working conditions or organisational issues.	16
	Nurses' Interactions with Older People in Need of Care		This category refers to narratives about the nurses' own or their colleagues' interactions with people in need of care over 80 years of age. This includes care, treatment, and communication.	4
Reputation of the Nursing Profession	Ideal Image of Nursing		This category refers to wishes for improvement in daily care practice and care in general. The ideal image of nursing refers to general needs in daily practice, desirable character traits of nurses needed in nursing, nursing actions needed in this age group (e.g. health promotion), or to nurses' communication skills.	26
	Desire to Enhance the Profession		Nurses express the wish to enhance the nursing profession or make explicit suggestions regarding how this can be achieved. Enhancing the profession means that the profession is strengthened, the reputation is increased, and the education is enhanced.	7
	Opinions about Nursing	Positive Opinions	Nurses report their positive opinions on caring for people over 80 years of age. This refers explicitly to care and care practices from the carer's perspective and not to attitudes towards older people in general.	36
		Negative Opinions	Nurses report their negative opinions about caring for people over 80 years of age. This refers explicitly to care and care practices from the carer's perspective and not to attitudes towards older people in general. It does not refer to inadequate working conditions but to facts that relate to the care of people in need of care, i.e. interaction with the patient.	24
	Career or Departmental Change Considered		Nurses indicate that they are thinking about, planning, or have already made a career change or changed their area of specialisation. This category also includes examples from participants who report that colleagues are considering, planning, or have already taken this step.	5
	Occupation not Conceivable or not Satisfactory		Nurses state that they cannot imagine working with people in need of care over 80 years of age or that they work with this group of people in need of care but find the work exhausting, frustrating, or unsatisfactory.	16
Criticism of Current Nursing Practice	Change in Nursing Stagnates		This category includes narratives about changes in care not being achieved, stagnating, or not being initiated.	2
	Inadequate Working Conditions	Structural or Institutional Grievances	This category refers to general reports of inadequate working conditions or conditions that are structural or institutional.	18
		Lack of Time	Nurses report not having enough time during their daily work.	20
		Role of Politics	Participants report on the role of politicians regarding the inadequate working conditions. This includes criticism of policy actions or reactions or the failure to do take action or react. Recommendations to policymakers also fall into this category.	6
		Shortage of Staff	This category includes narratives that identify staff shortages as contributing to inadequate working conditions.	26
		Too Little Payment	This category includes narratives that identify low pay as contributing to inadequate working conditions.	9
	Desire for Change in the Future for oneself		Nurses say that they hope the care and setting will have changed when they are older and need care. Worries about the future also fall into this category.	8
	Physical and Psychological Effects of Care on Nurses		Participants report on the physical and psychological effects of working with people in need of care over the age of 80 on themselves, or their concerns about this.	12
	Wish for Other Tasks		Participants' statements that they are unable to do the work they should or want to do fall into this category. Nurses' wishes for different task assignments also fall into this category.	2
	Education or Specialisation of Nurses		Nurses state that, in practice, further training or specialisation for nurses is lacking or desired.	12

### 3.1 Opinions on older people in need of care

Seven subthemes were identified within the overarching theme of ‘opinions about older people in need of care’, with the most narratives (16) assigned to the subtheme ‘dealing with older people in need of care is difficult or demanding’.

#### 3.1.1 Neutral opinions about older people in need of care

‘Neutral opinions about people in need of care’ were expressed by nurses who stated that care dependency does not depend on age or that the number of older people increases, thus increasing the number of people in need of care in nursing and care facilities. One respondent remarked that ‘Members of any age group can have a need for care. I find it strange to equate the need for care with age’ (SN 237, 60 years, no setting). Another respondent noted that older people in need of care do not always want to spend their time with other people; they simply want to spend some time alone. ‘[…] not every old person always wants to be with others. Some like to be alone […]’ (SN 83, 50 years, acute care).

#### 3.1.2 Negative opinions about older people in need of care

Regarding ‘negative opinions about people in need of care’, some nurses said that they perceived older people in need of care as aggressive, lazy, selfish, stubborn, and undiscerning. One respondent stated, ‘I react aggressively now when one older patient after another comes to the ward transported flat on their backs with the ambulance … back and forth 100 times with the movable toilet … hearing “Nurse can you … " or " ….can you make me …”’ (QN 185, 33 years, acute care). Some nurses also reported that older people in need of care were ‘deported’ to inpatient facilities or nursing homes and were incapacitated by our health care system. One person commented that ‘Unfortunately, in our society and health care system, people over 80 are disempowered, devalued, put in inpatient “reception camps”, dehumanised, and reduced to their mere bodily functions.’ (QN 601, 50 years, long-term care).

#### 3.1.3 Positive opinions about older people in need of care

The main observation made in the subtheme ‘positive opinions about people in need of care’ is that older people in need of care deserve respect, because of their life experience. Some respondents stated that they preferred people in need of care 80 years of age and older over younger people in need of care because they felt that they are thankful and straightforward. ‘I appreciate the experiences that older people have had.‘, commented QN 177 (39 years, acute care), noting that ‘This generation still experienced the war and is quite grateful and uncomplicated. This is in stark contrast with those under 80, who are completely different.’

#### 3.1.4 Nurses benefit from older people in need of care

Nurses reflected on the benefit they received from working with older people. Mostly, the respondents reported that they could learn a lot about themselves and their personalities by working with older people in need of care and learning about their life experiences. ‘I learn the most about myself while working with patients. How I would like to grow old and how not’, commented QN 85 (54 years, long-term care). Some respondents shared that older people in need of care had a calming effect on them due to their positive view of things. ‘I like to visit residents with dementia and chat with them. This can be relaxing, because they have their own positive view of things’ said QN 1626 (57 years, acute care).

#### 3.1.5 Dealing with older people in need of care is difficult or demanding

In the subtheme ‘dealing with people in need of care is difficult or demanding’, survey participants stated that caring for people over 80 years of age who are in need of care, although it is interesting and fulfilling work, is also demanding and exhausting. They also associated these challenging problems in nursing practice with conflicts between the nurse and patient. ‘[…] and that is why working with them is frustrating and also often leads to aggression among older people and conflicts’, commented QN 1420 (28 years, acute care). Several respondents stated that these challenges can arise from care needs related to underlying diseases, and especially diseases related to dementia. ‘Due to increasing delirium and dementia in older people, care is unfortunately becoming more and more difficult.‘, noted NA 1266 (41 years, acute care).

The involvement of older people's relatives was discussed by respondents as another challenging aspect in care. These respondents' narratives indicated that the relatives hampered the care of older people by expecting too much progress too quickly from the person in need of care.

The relatives are the ones who often make providing the care as part of the daily routine on the ward more difficult. The relatives are increasingly demanding and dissatisfied. Their expectations are often that these older people will be discharged back to home care in perfect health. Or they decide to transfer them to nursing homes without having discussed this with the patients themselves. Hardly anyone is willing to take over the care at home or to try it with an external offer of help. (QN 974, 47 years, acute care).

#### 3.1.6 Nurses’ interactions with people in need of care

In the subtheme ‘nurses’ interactions with people in need of care’, the communication between nurses and people in need of care is frequently described as lacking. The respondents noted that some nurses tended to label people in need of care who need more time to do something or to answer a question as having dementia. ‘I see how our young nursing staff communicate at the bedside every day,’ remarked QN 86 (58 years, acute care). ‘[I see them] not giving people time to understand what is being said.’ Respondents also stated that nurses can be too strongly focussed on theory and fail to use their common sense.

#### 3.1.7 Reputation of the nursing profession

In the theme ‘reputation of the nursing profession’, five subthemes were identified. Out of these narratives, 50.8% refer to ‘opinions about nursing’.

#### 3.1.8 Ideal image of nursing

The survey participants indicated that the ‘ideal image of nursing’ subtheme consists of nurses' opinions that nurses should have empathy, appreciation, respect, flexibility, and patience. ‘Nursing and caring for older people is a task that requires nurses to have a strong sense of responsibility, expertise, empathy, and flexibility’, commented QN 708 (55 years, long-term care). Some nurses noted that it is important that holistic care is provided, that people are allowed to age with dignity, and that they receive adequate palliative care. They also stated that it was important to them that nursing adapts to the patient with regard to the tempo. ‘They [the nurses] need to be made aware that they [the patients] have a different pace of life to which the daily care routine must adapt and not vice versa”, remarked QN 1436 (25 years, acute care). Some respondents also mentioned that the promotion of health and quality of life is also crucial in old age. ‘The quality of life should be preserved, especially in the last stages of life’ (QN 1436, 25 years, acute care).

#### 3.1.9 Desire to enhance the profession

Nurses see the need to enhance the status of the nursing profession and made three explicit suggestions for improvement. First, by raising the education to an academic level. ‘The nursing profession must be enhanced. An academic education [is needed]’, insisted QN 28 (32 years, acute care). Second, by using a better selection procedure for nursing students. ‘By now, everyone is accepted into the nursing programme. As in other health professions, only the best applicants should be taken (example of physiotherapy)’, remarked QN 185 (33 years, acute care). Third, by enabling a skill and grade mix in nursing practice. ‘Furthermore, I really hope that there will also be a real skill and grade mix in this sector due to the contribution of other professions, because nursing alone cannot do anything here’, commented QN 1217 (56 years, acute care).

#### 3.1.10 Opinions about nursing

The subtheme of ‘opinions about nursing’ was divided into two further subthemes: (1) ‘negative opinions about nursing’ and (2) ‘positive opinions about nursing’, the latter of which includes 60% of the narratives assigned to ‘opinions about nursing’.

With regard to ‘negative opinions about nursing’, survey participants reported that positive experiences with patients 80 years and older are rarer than with younger people in need of care. QN 100 (30 years, acute care) noted that ‘[…] the caregivers have significantly less frequent positive experiences than they do when caring for people in a younger age group’. Respondents also noted that the profession seems to have lost its sense of pride and prestige and that the work is getting more strenuous, frustrating, and difficult because older people stay at home longer and the number of people in need of care with psychiatric illnesses is increasing. ‘Care is becoming more and more complex because the residents can stay at home longer and longer due to the good care (home care) they receive’ (NA 570, 52 years, no setting). At the same time, respondents suggested that the appreciation for the profession has decreased, which also shapes their opinion of old people. QN 305 (43 years, long-term care) remarked that ‘You feel as though you are not worth anything, and neither is the work you do. And I think that shapes the image of the old person.’

With regard to ‘positive opinions about nursing’, some narratives described the work as interesting and worthwhile. ‘Caring for older people can be challenging, beautiful, and rewarding’ wrote QN 103 (34 years, long-term care). Respondents also stated that caring for older people is important work and that nurses doing that job deserve respect. Caring for older people was also described as meaningful work and as a supreme discipline in nursing. ‘Caring for older people is very important, no matter what stage of life you are in. It is worth every effort!’ (NA 1283, 25 years, acute care).

#### 3.1.11 Career or departmental change considered

Nurses commented that they were considering a career or departmental change. ‘I'm thinking about leaving nursing altogether before I get burned out’, noted QN 185, 33 years old and working in the acute care field. Some nurses reported that it was difficult to encourage the younger generation to keep working with older people. ‘It is difficult to encourage young people to stay in the nursing profession,’ remarked NA 45 (55 years, long-term care).

#### 3.1.12 Occupation not conceivable or not satisfactory

The respondents generally expressed the opinion that caring for older people is sad and frustrating work and that it is an undesirable occupational field. Several commented that it is frustrating for them that they cannot save every life and that more older people are overweight today, which complicates their work. Some of the nurses could not imagine continuing in this occupational field. ‘At present, the occupational field of gerontological care is relatively unattractive’, noted one respondent (no profession stated 863, 30 years, acute care). Another respondent stated that they did not feel it is possible for them to work in a way that is satisfactory for them anymore. ‘This results in a warm-full-clean-care routine, which, in turn, is frustrating for the nursing staff’, said SN 1019 (43 years, acute care).

So long as we always have the feeling that we have not finished, that we have not cared for people properly because the structure does not allow it, and that we are totally exhausted and rush out of the facility 20 minutes after the end of the shift, no one will recommend the profession to others. (SN 829, 42 years, acute care)

#### 3.1.13 Criticism of current nursing practice

Six subthemes could be identified that were assigned to the theme ‘criticism of current nursing practice’. The highest number of narratives (69.6%) refer to ‘inadequate working conditions’.

#### 3.1.14 Change in nursing stagnates

In the subtheme ‘change in nursing stagnates’, respondents said that many people talk about change, but change does not happen, which saddens and shocks them. ‘There is always a lot of talking and writing about improvement in this area, but nothing has changed. Which actually makes one very sad and bewildered’, commented NA 1358 (55 years, no setting).

#### 3.1.15 Inadequate working conditions

Inadequate working conditions were categorised into five subthemes: (1) ‘structural or institutional grievances’ (22.8%), (2) ‘lack of time’ (25.3%), (3) ‘role of politics’ (7.6%), (4) ‘shortage of staff’ (32.9%), and (5) ‘too little payment’ (11.4%).

In the subtheme (1) ‘structural or institutional grievances’, nurses criticised the working conditions, stating that these made their work frustrating rather than the older people. Due to inadequate working conditions, many nurses found it difficult to provide what they felt was adequate care for older people, which then made it difficult for them to help the older people age in dignity. ‘It's just that the conditions in our profession have become such that it is often no longer possible to work the way you would like to’, emphasised QN 928 (40 years, acute care). The health care system infrastructure was criticised, with respondents noting that it did not enable them to fulfil the everyday wishes and needs of older persons. They also remarked that they a too great focus was placed on documentation. Overall, the respondents had difficulties maintaining a healthy work-life balance. Many noted that they felt as though the institutions did not provide enough incentives to retain their staff and focussed too strongly on economic aspects. Some nurses cited grievances in the accommodations for older people, like SN 83 (50 years, acute care):

I recently had a frightening experience. I was told that, for example, the apartments in an assisted living unit are only renovated to a very minor extent, because they [the patients] are constantly coming and going, and you can't invest that much in them.

Some nurses also remarked that they felt as though care for nurses as well as people in need of care was needed, noting that this leads to frustration and sometimes aggression on both sides. They voiced criticism of the fact that institutions did not seem to be preparing for the demographic changes and the accompanying increase in patients with diseases like dementia. ‘Particularly in the long-term care sector, there is constant coercion on the part of both the person in need of care and the caregiver. This leads to frustration and sometimes aggression on both sides’ said QN 169 (50 years, long-term care).

Regarding the subtheme (2) ‘lack of time’, some respondents stated that they felt as though they were under time pressure at work and noticed the overall lack of time (for tasks). Due to this time pressure, they stated that they had no to little time to deliver the care tasks. They wrote that they found it difficult to provide individualised care and support for older people and only the most important work can be done.

[…] unfortunately, there is little or no time for the clients/patients, and often only the most important activities can be carried out. The staff has long since run out of time for promoting health promotion or holding simple conversations. Unfortunately, this applies to all areas/wards/facilities in our health care system. (SN 1408, 39 years, acute setting)

Several nurses also commented that the time pressure reduces the attractiveness of the occupation. ‘If nursing had time for care again, the job would be quite desirable,’ remarked SN 829 (42 years, acute care).

Another point of criticism arose in the subtheme (3) ‘role of politics’. Some nurses criticised the fact that, instead of making the occupation more attractive by reducing working hours or increasing wages, people were being pushed into the care profession and receiving the impression that anyone can provide care. These respondents worried that this might affect the image of geriatric care. ‘Above all, politicians would have to redefine the basic conditions for nursing staff. Create a balance between attractive pay and attractive working conditions … ‘, said SN 192 (36 years, long-term care). They demanded that the number of required staff should increase nationwide and that it should be mandatory. Furthermore, some nurses expressed a wish for a nursing care reform and for politicians to begin rethinking nursing and initiate change. ‘Rethinking our government and thus also carrying out actions would be appropriate. Simply applauding the nursing professions helps no one, neither the staff nor the patients/clients’ (SN 1408, 39 years, acute care).

In the subtheme (4) ‘shortage of staff’, some nurses commented that there were too few staff and that key staff were restricted to caring for older people, leading them to find the work frustrating. These respondents indicated that they did not have the resources and energy to provide individualised, adequate, and professional care. ‘[…] more staff - overworked nurses cannot give older people what they deserve! Some try, but they end up in burn-out!’ (QN 1661, 40 years, acute care). Moreover, the shortage of staff and resulting difficult working conditions reduce the attractiveness of the occupation. Many respondents remarked that the conditions are ‘[…] inhumane on both sides [nurses and those being cared for]’ (SN 191, 44 years, acute care). Some remarked that the coronavirus pandemic had worsened the situation. ‘The COVID-19 pandemic has additionally contributed its mite to the fact that the staffing situation has become very tense. Overtime is not the exception; it is the rule!’ said QN 501 (47 years, long-term care).

The last point of criticism in the subtheme (5) ‘inadequate working conditions’ is ‘too little pay’. Many nurses argued that, because of the low pay, working in long-term care is not desirable and that a wage increase would make working in care a more interesting career choice. ‘Only the pay […] prevents me from working directly in nursing homes (worked in a nursing home for 2.5 years a few years ago)’, revealed QN 459 (53 years, acute care).

#### 3.1.16 Desire for change in the future for oneself

Some nurses expressed their hope that they would see a change in the future when they were older and in need of care themselves. They also named explicit points where they would like to see this change: more appreciation for older people, more staff working in nursing homes, and overall more budget for the health care system. Some respondents expressed their fear of getting old and needing care because they thought that the situation of nursing would be worse in the future. ‘What really scares me is becoming […] frail myself and, even worse, getting dementia. The staffing ratio is getting tighter, the finances tighter. What does the future look like for us?’ (NA 232, 62 years, no setting).

#### 3.1.17 Physical and psychological effects of care on nurses

In the subtheme ‘physical and psychological effects of care on nurses’, some nurses described health consequences they had experienced or were afraid of experiencing because of their work in nursing. On the one hand, these respondents had experienced or feared physical illnesses due to the great physical strain. SN 1006 (53 years, acute care) commented that ‘[…] but it [working with older people] physically broke me.’ On the other hand, some reported that they had suffered or feared suffering psychological illnesses like burn-out. ‘Personally, I can't imagine that you can really stand it until you retire. I think you already will reach the end of your rope physically and mentally much sooner’ (NA 570, 52 years, no setting).

#### 3.1.18 Wish for other tasks

Many survey participants expressed their wish to do other tasks. These respondents commented that they had the feeling that they were not using their full potential and did not have the opportunity to apply what they had learned. ‘The whole morning I'm only busy with personal hygiene, I wouldn't have needed a diploma training’, remarked QN 185 (33 years, acute care). Some nurses noted that they took over the duties of people in other professions and, thus, had no time for nursing activities. SN 829 (42 years, acute care) expressed this concern, noting that nurses do not perform activities of nurses: ‘At present, nursing is being a cleaner, a secretary, a resident physician, a patient transporter, an auxiliary worker, etc.’

#### 3.1.19 Education or specialisation of nurses

In narratives assigned to the subtheme ‘education or specialisation of nurses’, some survey participants stated that one cannot stay in geriatric care for long without receiving further education. These respondents noticed that a certain technical knowledge and social skills were lacking. Nurses who made comments assigned to this subtheme demanded the opportunity to specialise in geriatric care, expressing a wish for education that focussed more strongly on the care of older people, on long-term care instead of acute care, and on illnesses relevant to the care of older people. They expressed the belief that improving education in this way would also improve the reputation of geriatric nursing. ‘Knowing that the proportion of older people in our society is increasing, nursing education should be adapted to this, i.e. much more knowledge about what dementia/delirium care means, and especially in acute hospitals!’ (QN 1612, 56 years, acute care). These respondents criticised the fact that it seemed difficult to obtain specific further education on care for older people unless they educated themselves. ‘We need well-trained, motivated, and committed people who will finally put the “bad” reputation of geriatrics in the right light’, said QN 1217 (56 years, acute care).

## 4 Discussion

This study was carried out to collect information about nurses' experiences and opinions on caring for people 80 years of age and older. The results of the qualitative analysis enabled us to categorise the nurses' narratives on nursing for older people into three main themes: their (1) ‘opinions on people in need of care’, their views on the (2) ‘reputation of nursing profession’, and their (3) ‘criticism of current nursing practice’. In each of these themes, nurses reflected on ‘positive opinions on nursing’, the ‘ideal image of nursing’, and the ‘shortage of staff’ most often.

‘Positive opinions on nursing’ was a subtheme associated with the highest number of narratives. This result shows that nurses value and consider their work with older people as important. In support of the findings of Özdemir and Bilgili 2016 [21], who listed reasons for nursing students to consider working with older people, the respondents appreciated the high importance of their work, indicating that they felt they were increasing the quality of care for older persons in need of care, and especially in the last phase of life. A positive attitude towards older people [18,19] and their care [22] was also identified as a reason that made survey participants decide to pursue a career in gerontological nursing. These participants reflected on their positive attitude towards people 80 years of age and older who were in need of care. These individuals also expressed appreciation for the life experience that older people have and indicated that they deserve respect. These findings agree with the core elements of the nurse-patient relationship, i.e. respect and dignity, cited by Riviere et al., 2019 [31]. Some nurses, however, described working with people 80 years and older as difficult and demanding due to their underlying diseases, e.g. dementia. These respondents stated that positive experiences occur less often than they do when caring for younger people in need of care. This result supports other results found in the international literature on geriatric nursing, which indicate that it is an undesirable career choice because this work is perceived as difficult due to the greater and more complex care needed by people in this age group [21].

Some nurses also reported that they were not satisfied with the current working conditions, describing experiences of ‘structural or institutional grievances’, a ‘lack of time’ for providing care, a ‘shortage of staff’, ‘too little pay’, and too little support from politicians. These factors have been shown to influence nurses' job satisfaction [53] and can cause them to leave their workplace or the profession [53,54]. Lower job satisfaction may lead to lower job performance and burn-out [53]. This was also mentioned by our study participants, who stated that they had experienced or feared experiencing the physical or psychological consequences of working as a nurse with older people in need of care.

Other nurses remarked that they were not satisfied with their own work performance, as they were often only able to provide basic care instead of individualised, high-quality care. This phenomenon is called ‘missed nursing care’, i.e. necessary care that is either wholly or partially omitted or delayed [55]. The meta-review on missed nursing care by Chaboyer et al., 2021 [56] shows that this is influenced by staffing levels, the staff skill mix, teamwork, lack of resources (e.g. materials), and the high number of administrative, non-nursing tasks. Our study participants also criticised the staff shortage, the lack of a skill-and-grade-mix, and the high number of administrative, non-nursing tasks they had experienced or observed. In their narratives, some nurses also noted that promoting autonomy, educating people in need of care, offering fundamental physical care, as well as emotional or psychological support tasks are neglected, among others [56]. This leads to a poorer quality of care and to lower job satisfaction [56], which seems to create a vicious circle.

In addition, some nurses in our study commented that the COVID-19 pandemic had increased the inadequacy of the working conditions described above. This study was performed at the end of the third COVID-19 wave. This might have influenced the nurses’ experiences of and opinions about caring for older people because this was a stressful and busy time for nurses. During the COVID-19 pandemic in hospitals in Europe and the USA, 53.1% of surveyed nurses experienced burn-out, and 49.1% rated their hospital negatively regarding patient safety. These nurses reported that their work was frequently interrupted due to staff shortages and the need to perform non-nursing tasks [57]. Austrian nursing staff working in nursing homes also reported that, although they had more time to care for residents due to cancelled therapies and the lack of visitors, they also had to deal with an additional workload due to hygiene measures, the need to take over tasks from other occupational groups (i.e. activity coordinators), and the fact that residents needed more attention, encouragement, and reminders to comply with the hygiene measures [58].

### 4.1 Strengths and limitations

The primary focus in this study was to measure nurses' attitudes towards people 80 years of age and older and their opinions about caring for them using a quantitative method [30]. We collected survey participants’ narratives in an open-ended comment box and decided to analyse these separately due to the extensive and high number of such narratives. This may represent a study limitation. The survey participants had to answer the quantitative survey questions on the questionnaire, which directed their attention towards their attitudes about and care for people 80 years of age and older, before they provided a response in the open-ended comment box. Because the nurses were made aware of attitudes and ageism beforehand, this may have influenced their narratives. Although many nurses completed the questionnaire, we had no influence over how many nurses provided narratives; therefore, we do not know if data saturation was reached. Furthermore, there is the possibility of a response bias. Participants who feel strongly about caring for older adults may have been more eager to give a narrative. Answers to general open-ended questions at the end of a questionnaire also tend to be more negative and critical narratives [59]. The distribution between the nursing professions was also random. Therefore, 81% of the collected narratives were made by nurses with a diploma and specialised nurses, and 61.1% by nurses in acute care. Nurses without a diploma and from long-term care or home care are underrepresented in this study. Therefore, we were not able to separately analyse the narratives of qualified nurses and nursing aides. When looking at differences in the characteristics of those who provided a comment and those who did not, no differences could be detected. Therefore, we concluded that this study has no motivational bias. As some settings and nursing roles are underrepresented in this study, we cannot make any statements on the transferability of these findings to all nursing settings and roles. Furthermore, about 20 % of the participating nurses worked in management, research or teaching and not directly with patients. As they are trained nurses and as they offer an additional angle of view which helps to obtain a more complete picture, we decided to include the narratives of these participants in this study. We did not ask a specific question when encouraging the participants to enter a narrative in the open-ended comment box. This might constitute a strength of this study, as we gave the participants the opportunity to freely express their opinions of older people and their care. Furthermore, this study was planned and executed as a cross-sectional study. Initially, it was not intended to analyse the narratives in the commentary box. Therefore, in the planning and execution of this study, no considerations were made with regard to qualitative rigour. Nonetheless, this study led to new themes with respect to caring for older persons, which was not the primary focus aim and which would not have emerged in a quantitative study. Another study limitation is that the answers provided by the respondents were written and not provided orally, e.g. in a face-to-face interview, where we would have had the opportunity to ask further questions on points raised by the participants.

### 4.2 Relevance to clinical practice

In order to encourage nurses to start working or to remain in geriatric care, we recommend that further education is offered, such as gerontological courses or specialised training for nurses or nursing students [60]. These were requested by our participants. Our results indicate that strengthening nurses' positive attitude towards older people and their care can increase their willingness to work with older people [18,19,22]. This can be achieved by introducing gerontological educational interventions for nurses and encouraging intergenerational contact [61]. These measures also emerged in nurses' narratives that reflected a negative view on older adults. Offering special training to improve teamwork might improve nurses' job satisfaction [60], and this may also prevent ‘missed nursing care’ as well as increase nursing staff [62]. In turn, improving the work environment and increasing nursing staff may result in an improvement in the quality of care [63]. For these reasons, we recommend policymakers and nursing managers to improve the working environment by increasing nursing staff, increasing pay, reorganising daily work (i.e. by reducing administrative tasks), and by preparing for the sociodemographic change. All of these points were mentioned by the participants of this study.

Further research is needed to investigate nurses' needs and wishes related to the care of people 80 years of age and older. Qualitative studies should also be performed, such as interviews with nurses from different settings and in different nursing roles. Furthermore, researchers should interview people in need of care who are 80 years of age and older to investigate their care needs and to ask them to describe their view of ‘ideal’ care. Participatory action research could be applied as an approach for improving the daily work of nurses.

## 5 Conclusion

Our study results show that nurses appreciate working with people of 80 years of age and older and perceive this work to be meaningful and important, but it reveals aspects that diminish their joy in their work. For instance, caring for older people can be difficult or demanding, and the surveyed nurses generally perceived the working conditions as inadequate. Further research is needed in the form of qualitative studies with nurses, and improvements must be made by policymakers and nursing managers (e.g. more education and nursing staff) to improve these working conditions. Nurses also described the poor reputation of geriatric nursing care in the eyes of the public and their colleagues. By sensitising nurses to the aspirations and joys of geriatric nursing and by offering further education and opportunities for specialised training, the image of geriatric nursing might improve.

## Ethics statement

This study was approved by the Ethics Committee of the Medical University of Graz (EK Number 31–320 ex 18/19). Participation was voluntary and could be cancelled at any moment. After reading the written information about the study, participants provided consent by answering 'yes' to the question of whether they wanted to participate. No conclusions could be drawn regarding the participants’ identity.

## Data availability statement

Data associated with this study have not been deposited into a publicly available repository. Data are not available due to the sensitivity of the written statements. The used data are confidential.

## CRediT authorship contribution statement

**Lena Maria Lampersberger:** Writing – review & editing, Writing – original draft, Methodology, Investigation, Formal analysis, Data curation, Conceptualization. **Gerhilde Schüttengruber:** Writing – review & editing, Writing – original draft, Investigation, Formal analysis. **Christa Lohrmann:** Writing – review & editing, Writing – original draft, Supervision, Conceptualization. **Franziska Großschädl:** Writing – review & editing, Writing – original draft, Supervision, Conceptualization.

## Declaration of competing interest

The authors declare that they have no known competing financial interests or personal relationships that could have appeared to influence the work reported in this paper.
